# Quality of Life, Hopelessness, Impulsivity, and Suicide in the Rural Elderly in China: A Moderated Mediation Analysis of Psychological Autopsy Data

**DOI:** 10.3389/fpubh.2022.900156

**Published:** 2022-06-16

**Authors:** Guoxiang Chen, Qiqing Mo, Xinguang Chen, Bin Yu, Huiming He, Guojun Wang, Cunxian Jia, Liang Zhou, Zhenyu Ma

**Affiliations:** ^1^Guangxi Medical University, Nanning, China; ^2^Guilin People's Hospital, Guilin, China; ^3^Department of Epidemiology, University of Florida, Gainesville, FL, United States; ^4^Institute of Parasitic Disease Control and Prevention, Guangxi Zhuang Autonomous Region Center for Disease Control and Prevention, Nanning, China; ^5^Shenzhen Graduate School, Peking University, Shenzhen, China; ^6^School of Public Health, Shandong University, Jinan, China; ^7^The Affiliated Brain Hospital of Guangzhou Medical University, Guangzhou, China

**Keywords:** suicide, quality of life, hopelessness, impulsivity, moderated mediation

## Abstract

**Background:**

People who had died by suicide always being associated with negative emotions and even mental disorders. Understanding mechanisms underlying the association between quality of life (QOL), hopelessness, and suicide are of great significance. In this study, we aimed to test a model in which the QOL-suicide relationship was mediated by hopelessness and moderated by impulsivity.

**Methods:**

Participants (*N* = 484, including 242 suicide deaths and 242 matched controls) were rural residents 60 years of age and older, randomly selected from 12 rural counties in China using a two-stage stratified cluster sampling method. Data were collected with standard psychological autopsy technique from informants (*n* = 968). The outcome variable was a suicide death. QOL, hopelessness, and impulsivity were assessed using validated scales. The proposed relationships were tested using mediation and moderated mediation models.

**Results:**

Of the total sample, 55.8% were men with a median age of 75.5 years. Results from the moderated mediation analysis indicated that QOL was negatively associated with suicide (beta = −0.141, *p* < 0.01); this association was mediated by hopelessness (indirect effect: beta =0.578, *p* < 0.01), accounting for 73% of the total effect. Impulsivity significantly moderated the mediation effect from QOL to hopelessness (beta =0.005, *p* < 0.01).

**Conclusions:**

Study findings have confirmed the negative association between QOL and suicide with psychological autopsy data, and demonstrated the role of hopelessness in mediating the QOL-suicide relation that is further modified by impulsiveness. These findings depend on our understanding of the suicide epidemiology among the elder in rural China and provide information much needed for suicide prevention.

## Introduction

Suicide is one of the most important public health challenges across the world, (1, 2) including in China (3). China has experienced a dramatic decline in suicide mortality per 100,000 from 17.6 in 1987 to 7.46 in 2014 (4). However, large disparities exist with the rate of suicide much higher for the elderly living in rural areas than in others (5). The high suicide rates underscore the need for investigating modifiable factors that are associated with suicide among the elderly in rural China as well as mechanisms underpinning the relationship between risk factors and suicide.

A number of studies have identified a series of bio-psycho-social factors for suicide, and a significant one is quality of life (QOL) (6). QOL is generally referred to as a positive sense of an individual's physical, psychological, and social well-being in the current social context (5). Understanding the impact of QOL on suicide among the elderly in rural China is of particular significance. QOL has often been linked to suicidal behaviors in various countries (6, 7), including China (8, 9).

Since the economic reforms in the 1980s, a large number of rural young people in China have left their homes and moved to urban areas while vulnerable elderly remain in rural areas waiting for assistance. Consequently, reductions in quality of life and increases in hopelessness may expose the rural elderly to an increased risk of suicide. The elderly in rural China may suffer from large reductions in QOL, including declines in the standard of living, and increases in many challenges, such as safety and social separation. In addition to suicidal behaviors, QOL has been directly related to suicide death in other populations (10).

Hopelessness is a negative cognition of one's future (11). Empirical studies have indicated that hopelessness is a warning sign, often prior to or co-existing with suicidal behaviors, and immediately prior to suicide death (12). Furthermore, hopelessness has also been empirically associated with suicidal behaviors (13, 14) and death (15) in other populations. A 10-year cohort study reported that hopelessness was reliable to predict the occurrence of suicide attempts among patients with psychosis (13).

Studies summarized in the previous sections indicate that QOL and hopelessness both are likely to be associated with suicide. Relative to QOL, hopelessness is a more proximal predictor (16); furthermore, documented studies indicate that QOL can significantly predict hopelessness (14), suggesting a potential role of hopelessness in mediating the QOL-suicide relationship. Empirical studies also indicate that hopelessness can mediate the relationship between perceived QOL and suicidal behaviors (17), but no study has examined the mediation mechanism by which hopelessness may mediate the QOL-suicide death relationship.

The impression has been arising that temperament or personality characteristics are associated with suicidal risk (18). Impulsivity is an important personality trait characterized by reflection impulsivity, impulsive action, and impulsive choice (19). Individuals with the personality of impulsivity are more likely to take risk behaviors, including suicide (20, 21). To test the proposed mediation mechanism that links QOL and hopelessness to suicide, the impact of this personality trait must be considered. This is because, with the same QOL level, individuals with higher levels of impulsivity may be more likely to become hopeless and more likely to commit suicide. Similarly, with the same level of hopelessness, the more impulsive individuals may be more likely to commit suicide than the less impulsive individuals. No reported study has examined these moderation effects.

**Figure 1:**
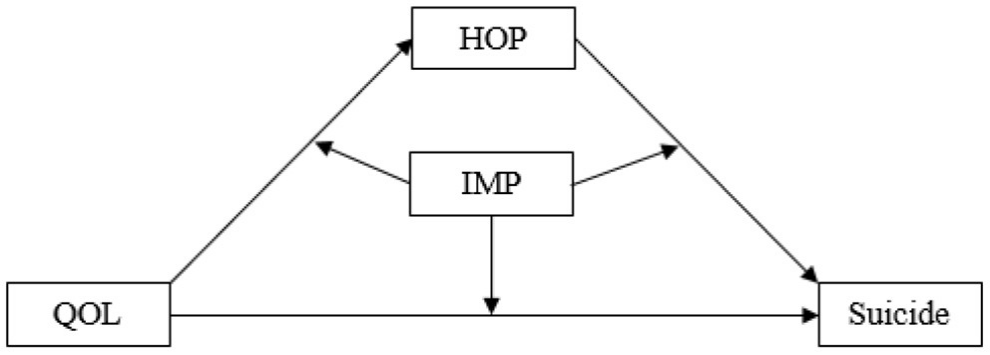
A proposed moderated mediation model that links quality of life with suicide through hopelessness, modified by impulsivity. QOL, the quality of life; HOP, hopelessness; IMP, impulsivity.

Few studies have examined the complex mechanisms underpinning the relationship between QOL, hopelessness, impulsivity, and suicide. With psychological autopsy data, this study tested proposed models in which hopelessness mediated the relationship between QOL and suicide death that was modified by impulsivity (Figure 1). The ultimate goal is to provide evidence supporting future prevention interventions targeting the elderly living in rural China for suicide reduction.

## Materials and Methods

### Participants and Sampling

This study is an analysis of data collected. A detailed description of participants and sampling has been reported elsewhere (22). A two-stage stratified cluster sampling method was used in our study. These provinces were selected based on GDP ranking from the top 10 (Shandong), 11–20 (Hunan), and 21–31 (Guangxi). Of the selected province, a total of 12 counties were randomly selected after ranking individual counties of a province by annual income, including three counties from Shandong, three from Hunan, and 6 from Guangxi.

Suicide deaths (cases) were identified using information from the death certificates available from the Center for Disease Control and Prevention at the county level. Participants were those with a formal record of suicide death reported by trained village doctors and local public health professionals, aged 60 years and older, and who committed suicide between February 2014 and September 2015. For each suicide death, a living control was selected through matching age (±3 years), gender, and geographic location (living in the same community). Whenever a suicide case was identified, the investigators would list and enumerate all candidates that matched. Then one living comparison was randomly selected from the list using a computer program. In a few cases when there were no appropriate living comparisons available, the investigators expanded the search to the nearest villages.

### Psychological Autopsy

Data were collected using the standard psychological autopsy technique (22). For each participant, two informants were selected. For each participant, two informants who were a family member and a friend or a neighbor, or other relevant people were selected to obtain the data related to them.

Data collectors were trained faculty and graduate students from three universities located within the selected provinces, including Shandong University, Central South University, and Guangxi Medical University. Data were collected through face-to-face interviews with an average interview time of 90 min.

The study was approved by the IRB of Shandong University, Central South University, and Guangxi Medical University.

### Measurements

Quality of life was measured using the Quality of Life Scale previously tested among Chinese in a psychological autopsy study (10). The scale consists of 6 items, assessing (a) physical health, (b) mental health, (c) economic circumstances, (d) work relationships, (e) family relationships, and (f) relationships with non-family associates. The time range was a month prior to suicide (cases) or the investigation (control). Scale items were assessed using a five-point Likert scale varying from 1 (*very bad*) to 5 (*very good*). The reliability and the validity have been reported in our previous study (23). Total scores were computed such that larger scores indicated a higher level of quality of life.

Hopelessness was measured by a short version of the Beck Hopelessness Scale (24). The short version uses 4 items to assess the four key constructs of hopelessness, including expectancies of the future with (a) success, (b) darkness, (c) lack of opportunity, and (d) faith. Individual items were measured using another five-point Likert scale ranging from 1 (*absolutely yes*) to 5 (*absolutely no*). The Cronbach's α was 0.834 in this study. Total scores were computed such that larger scores indicated higher levels of hopelessness.

Impulsivity was measured using Barratt Impulsiveness Scale (Chinese version) (25). This 30-item scale assesses (a) impulsive planning, (b) motor impulsiveness, and (c) cognitive impulsiveness. Individual items were measured using another five-point Likert scale ranging from 1 (*never*) to 5 (*always*). The Cronbach's α was 0.913 in this study. Total scores were computed such that higher scores indicated more impulsiveness.

Demographic variables were gender (male/female), age (in years), stability of marriage (stable vs. unstable), an education level (less than primary school/primary school/more than primary school), employment (employed/unemployed/retired), family annual income (RMB Chinese yuan), physical disease (y/n), mental disorder (y/n), and if living alone (y/n).

### Statistical Analyses

Descriptive statistics were used to describe the study sample. Correlation analysis was used to explore the relationship between suicide and other factors, including quality of life, hopelessness, and impulsivity, as well as demographic and social-economic status. A moderated mediation model was proposed and used in investigating the mechanism by which the perception of hopelessness in the mediation of the relationship between quality of life and suicide, is moderated by impulsivity (Figure 1). The SAS macro PROCESS developed Hayes (26) was used to assess the moderated mediation model. Type I error was set at *P*-values < 0.05 level for statistical inference. All statistical analysis was conducted using the commercial software SAS, version 9.4 (SAS Institute Inc, Cary, NC, USA).

## Results

### Study Participants

Table 1 summarizes the main characteristics of the study participants. Of the total sample, 242 were cases and 242 controls with a median age of 75.5 years and 55.8% men. Among the participants, 42.8% had less than primary education, and 33.5% had a family annual income of <3,600 Chinese yuan. Relative to the controls, the suicide cases were more likely to be left-behind with an unstable marriage, lived alone, unemployed, and diagnosed with physical and mental disorders.

**Table 1:** Demographic characteristics for the study subjects.

**Variable**	**Suicides**	**Controls**	**Total**	**χ^2^/t**	***P***
Total, *n* (%)	242 (50%)	242(50%)	484 (100%)		
Gender, *n* (%)					
Male	135 (55.8)	135 (55.8)	270 (55.8)	–	–
Female	107 (44.2)	107 (44.2)	214 (44.2)		
Age (in years) [Median (QR)]	75 (68, 81)	76 (67.75, 80)	75.5 (68, 80)	0.511	
Education, *n* (%)					
Less than primary school	111 (45.9)	96 (39.7)	207 (42.8)	1.920	0.383
Primary school	105 (43.4)	116 (47.9)	221 (45.7)		
More than primary school	26 (10.7)	30 (12.4)	56 (11.6)		
Marriage stability, *n* (%)					
Stable	122 (50.4)	170 (70.2)	292 (60.3)	19.890	<0.001
Unstable	120 (49.6)	72 (29.8)	192 (39.7)		
Being left-behind, *n* (%)					
Yes	41 (16.9)	25 (10.3)	66 (13.6)	4.491	0.034
No	201 (83.1)	217 (89.7)	418 (86.4)		
Employment, *n* (%)					
Employed	40 (16.5)	59 (24.4)	99 (20.5)	7.837	0.021
Unemployed	195 (80.6)	169 (69.8)	364 (75.2)		
Retired	7 (2.9)	14 (5.8)	21 (4.3)		
Family annual income (Chinese yuan, RMB), *n* (%)					
0–3,600	88 (36.4)	74 (30.6)	162 (33.5)	1.865	0.394
3,601–10,000	88 (36.4)	98 (40.5)	186 (38.4)		
above 10,001	66 (27.3)	70 (28.9)	136 (28.1)		
Living alone, *n* (%)					
Yes	64 (26.4)	35 (14.5)	99 (20.5)	10.679	0.002
No	178 (73.6)	207 (85.5)	385 (79.5)		
Physical diseases, *n* (%)					
Yes	202 (83.5)	161 (64.9)	363 (75.0)	18.523	<0.001
No	40 (16.5)	81 (35.1)	121 (25.0)		
Mental disorders, *n* (%)					
Yes	122 (50.4)	12 (5.0)	134 (27.7)	124.870	<0.001
No	120 (49.6)	230 (95.0)	350 (72.3)		

### Correlations Between Suicide and Other key Variables

Results in Table 2 indicate that suicide was significantly correlated with QOL (*r* = −0.55, *p* < 0.01) and hopelessness (*r* = 0.68, *p* < 0.01), and QOL and hopelessness was also correlated (*r* = −0.71, *p* < 0.01), suggesting a potential role of hopelessness in mediating the QOL-suicide relationship. In addition, impulsivity was significantly correlated with QOL (*r* = −0.5, *p* < 0.01) and hopelessness (*r* = 0.49, *p* < 0.01) and suicide (*r* = 0.35, *p* < 0.01), suggesting potential roles of this variable in modifying the relationship between QOL, hopelessness and suicide.

**Table 2:** The correlation among the variables of the rural elderly.

**Variable**	**Median (QR)**		**Suicide**		**Sex**		**Age in year**	
			**Correlation coefficient**	***P***	**Correlation coefficient**	***P***	**Correlation coefficient**	***P***
Suicide	–		–					
Sex	–		–		–			
Age in year	75.5 (68, 80)		0.02	0.61	0.03	0.47	–	
Education	2 (0, 4)		−0.06	0.17	0.38	<0.001	−0.21	<0.001
Marriage	1 (1, 2)		0.2	<0.001	−0.15	<0.001	0.4	<0.001
Quality of life	17 (15, 20)		−0.55	<0.001	−0.09	0.06	−0.07	0.11
Hopelessness	12 (9, 15)		0.68	<0.001	0.06	0.22	0.05	0.31
Impulsiveness	92 (80, 105)		0.35	<0.001	−0.05	0.31	0.12	<0.001
**Variable**	**Education**		**Marriage**		**Quality of life**		**Hopelessness**	
	**Correlation** **coefficient**	***P***	**Correlation** **coefficient**	***P***	**Correlation** **coefficient**	***P***	**Correlation** **coefficient**	***P***
Suicide								
Sex								
Age in year								
Education	–							
Marriage	−0.16	<0.001	–					
Quality of life	0.04	0.35	−0.11	0.01	–			
Hopelessness	−0.05	0.25	0.15	0.001	−0.71	<0.001	–	
Impulsiveness	0.17	<0.001	−0.2	<0.001	−0.5	<0.001	0.49	<0.001

### Results From the Mediation Modeling Analysis

**Figure 2:**
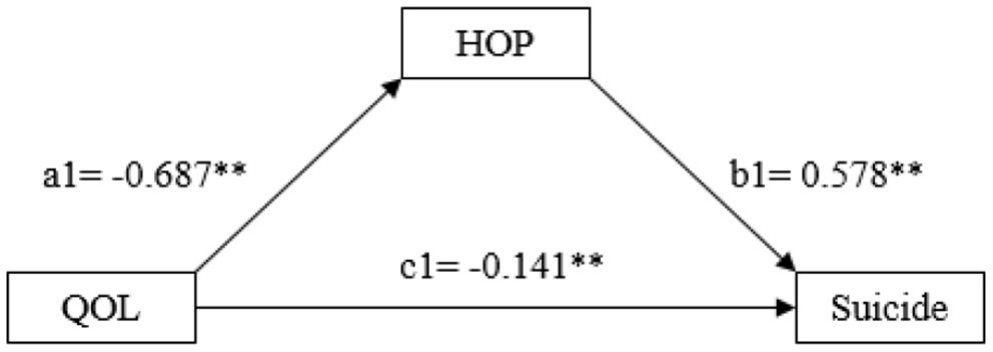
Schematic model of hopelessness as the mediator in the relationship between quality of life and suicide. QOL, quality of life, and HOP, hopelessness. Model coefficients were estimated using the SAS Macro PROCESS (see text for detail); *: *p* < 0.05, and **: *p* < 0.01.

Results in Figure 2 indicate that QOL was associated with suicide deaths through two paths, a direct effect (coefficient c1 = −0.141, *p* < 0.01) and an indirect effect (a1^*^b1 = −0.397) through hopeless with coefficient a1 = −0.687, *p* < 0.01 for the QOL – hopelessness relation and b1 = 0.578, *p* < 0.01 for the hopelessness-suicide relation. The total effect = indirect effect + direct effect = −0.141 + −0.379 = −0.52, with the indirect effect for 73% (0.379/0.52) of the total effect.

### Results From the Moderated Mediation Analysis

**Figure 3:**
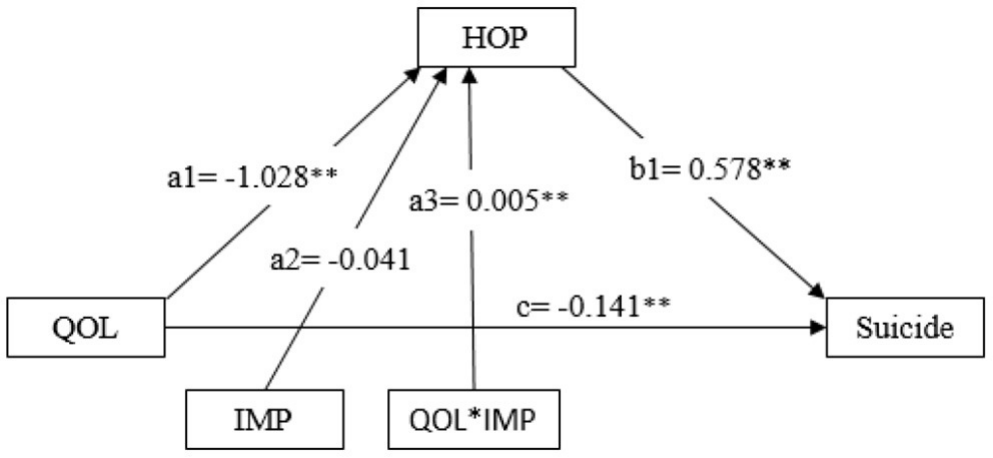
Schematic model of impulsivity as the moderator in the relationship between quality of life and hopelessness, quality of life and suicide. QOL, quality of life; HOP, hopelessness; IMP, impulsivity. Model coefficients were estimated using the SAS Macro PROCESS (see text for detail); *: *p* < 0.05, **: *p* < 0.01.

Of several moderated mediation models, a significant moderation effect was detected for only one model (Figure 3). The result in the figure indicates that impulsivity significantly moderated the QOL-hopelessness relationship with the model coefficient =0.005, *p* < 0.01, suggesting a stronger QOL-hopelessness relationship for individuals with a higher level of impulsivity.

## Discussion

In this study, the QOL-suicide relationship that is mediated by hopelessness and moderated by impulsivity has been tested with data collected through psychological autopsy among the rural elderly in China. Study findings provide valuable data deepening our understanding of the factors related to suicide among the elderly in rural China and informing evidence-based interventions for suicide reduction.

Quality of life is a modifiable and protective factor for suicide (10, 27). In this study, we confirmed the negative association between QOL and suicide among the elderly in rural China as reported in other populations within and outside of China (17). With quick economic growth and a large number of rural-to-urban migrants, more attention should be paid to rural elderly who may not benefit but suffer from the development. It is indicating that the quality of life for the rural elderly should be improved in the rapid economic development to reduce their risk of suicide.

A novel finding of this study is that a majority of the QOL-suicide relationship for the elderly in rural China is mediated through hopelessness. This means that although low QOL may increase suicide among the elderly in rural areas, this will not happen for most elderly if they still have hope in their life.

According to this finding, one approach to suicide prevention for the elderly in rural China would be to fight against hopelessness (28). Hopelessness is often resulted from the lack of perceived capabilities to cope with the challenges (29) and alleviation of hopelessness cognition may reduce suicide risk (30). Therefore, hopelessness can be reduced by the provision of assistance and training to solve problems, such as separation from family members, poor transportation, poverty, chronic diseases, and lack of access to healthcare. Another issue is that most Chinese elderly tend to solve problems by themselves rather than asking their children for help with the intention not to add extra burden to their children while their children may in fact want to help them. We must consider this factor in devising intervention programs to reduce the level of hopelessness among the elderly.

The findings of the study also indicate that impulsivity is a significant moderator that can alter the relationship between QOL and hopelessness. Compared to those with low levels of impulsivity, the elderly with a high-level are more likely to experience hopelessness and commit suicide. This may be because impulsivity is a personal trait affecting individuals' coping behaviors. People with a higher level of impulsivity tend to make decisions without rational and analytical thinking, thus, they are more likely to develop hopelessness (31). We must also consider this factor in devising programs for suicide prevention blocking the process from low QOL to hopelessness for the elderly in rural China.

There are limitations to this study. Firstly, the interview was held after suicide death 2–6 months, the information was collected from informants with recall bias. Secondly, there are methodological limitations in psychological autopsy studies. The use of proxy informants, the lack of blinding about suicide, and comparison have an impact on the reliability of the data. Thirdly, the study participants were selected using a two-stage stratified cluster sampling method, they were selected only from three provinces in China. There was its own cultural background in each region which has a special influence on people's cognition. Caution is needed when generalizing the findings of this study to the elderly population across China given the high heterogeneity in the elder population across the large area of China.

## Conclusion

In conclusion, this study is the first to apply a moderated mediation model in investigating three key influential factors, QOL, hopelessness, and impulsivity. Also, the first one is to investigate the mechanisms underpinning the complex relationship linking these factors to suicide among the elderly in rural China with psychological autopsy data. The findings of this study provide new data advancing our understanding of the epidemiology of suicide among the elderly and informing evidence-based interventions for suicide reduction. The quality of life should be improved and the hopelessness should be reduced to decrease the rate of rural elderly in China. What is more important is to reduce the risk of suicide among older people by improving their ability to deal with problems.

## Data Availability Statement

The raw data supporting the conclusions of this article will be made available by the authors, without undue reservation.

## Ethics Statement

The studies involving human participants were reviewed and approved by the study was approved by the IRB of the Central South University, Shandong University, and Guangxi Medical University. The aim and procedure of the research were explained to all participants. Written informed consent must be obtained before interviews were conducted. The patients/participants provided their written informed consent to participate in this study.

## Author Contributions

GC, QM, and XC conducted the analysis and drafted the manuscript. BY undertook the statistical analysis and revised the draft. HH managed the literature searches. GW participated in the data collection and data management. CJ, LZ, and ZM designed the study and wrote the protocol. All authors contributed to the article and approved the submitted version.

## Funding

This work was supported by the [American Foundation of Suicide Prevention #1] under Grant [number SRG-0-169-12]; [Natural Science Foundation of Guangxi Province #2] under Grant [number 2014GXNSFBA118163].

## Conflict of Interest

The authors declare that the research was conducted in the absence of any commercial or financial relationships that could be construed as a potential conflict of interest.

## Publisher's Note

All claims expressed in this article are solely those of the authors and do not necessarily represent those of their affiliated organizations, or those of the publisher, the editors and the reviewers. Any product that may be evaluated in this article, or claim that may be made by its manufacturer, is not guaranteed or endorsed by the publisher.
